# Performance Research and Formulation Optimization of High-Performance Local Insulation Spray Coating Materials

**DOI:** 10.3390/nano12193344

**Published:** 2022-09-25

**Authors:** Hechen Liu, Liwei Wei, Fengsheng Gao, Li Tang, Le Li, Zhanglin Sun, Yunpeng Liu, Peng Dong

**Affiliations:** 1 Hebei Key Laboratory of Green and Efficient New Electrical Materials and Equipment, North China Electric Power University, Yonghua North Street No. 619, Baoding 071003, China; 2 Electric Power Research Institute of China Southern Power Grid, Kexiang Road No.11, Guangzhou 510000, China

**Keywords:** polyurethane-modified epoxy resin, orthogonal test method, nano-Al_2_O_3_, nano-SiO_2_

## Abstract

Bird pest control has become a major task for the operation and maintenance of distribution network lines. Epoxy resin that cures quickly at room temperature can be used to coat locations where birds frequently build their nests. However, epoxy resin has enormous internal stress and is brittle, so it is essential to toughen it. In this paper, for a room temperature curing system composed of polyurethane-modified epoxy resin and a polythiol curing agent, three kinds of particles, i.e., Al_2_O_3_, SiO_2_, and Mg(OH)_2_, were used to modify a polyurethane modified epoxy resin. Orthogonal experiments were designed to study the effects of different fillers on the comprehensive properties of polyurethane-modified epoxy resins. The experimental results showed that there were not only independent effects of different kinds if particles on the resin, but also synergistic effects of multiple particles. Nanoparticles can reduce the defects introduced by microparticles to a certain extent and improve the mechanical and electrical properties of the resin. The overall performance of the resin was optimized when the amounts of SiO_2_, Al_2_O_3_, and Mg(OH)_2_ were 1.7%, 2.5%, and 7%, respectively. The tensile strength of the resin was increased by 70%, the elongation at a break by 67.53%, and the breakdown strength by 20.31% compared with before the addition of filler. The microscopic morphology and thermal properties of the resin before and after the addition of filler were also studied. Adding fillers caused more cracks to absorb part of the energy when the resin matrix was stressed and increased the rigidity of the resin matrix and the resin’s glass transition temperature (T_g_) by 13.48 °C. Still, the temperature corresponding to the maximum rate of weight loss (T_max_) remained unchanged.

## 1. Introduction

In recent years, birds have bred in large numbers with the gradual improvement of the environment and large-scale agricultural development. Coupled with the development and construction of industrial parks, leading to a reduction in trees, birds are prone to choosing to nest in line tension-resistant poles, line switches, cutters, line clamps, cross-arms, etc., which contain exposed conductive points and weak insulation connection parts, making them prone to short-circuit faults, which, in turn, can cause power outages, equipment burns, and other serious failures. As such, bird damage to transmission lines constitutes an increasingly significant hazard [1].

Numerous academics have examined the behaviors of various bird species and have made the following four significant findings [2,3,4]: (1) When birds nest on distribution line towers, wires or other metal flying shuttle wires, short circuit faults may occur; (2) Bird nests comprising branches, feathers and metal wires are easily wetted, and the resulting bare conductive points can cause interphase defects; (3) In exceptional weather conditions, bird droppings accumulate on insulators causing flashover faults along the surface; and (4) Birds pecking at composite insulator silicone rubber sheathing reduces insulation performance and service life. According to statistics, a total of 70 bird damage faults occurred in Hunan Province between 2006 and 2016; these included six trips involving bird nests, 61 trips involving bird droppings, and three involving bird bodies [5].

**Figure 1:**
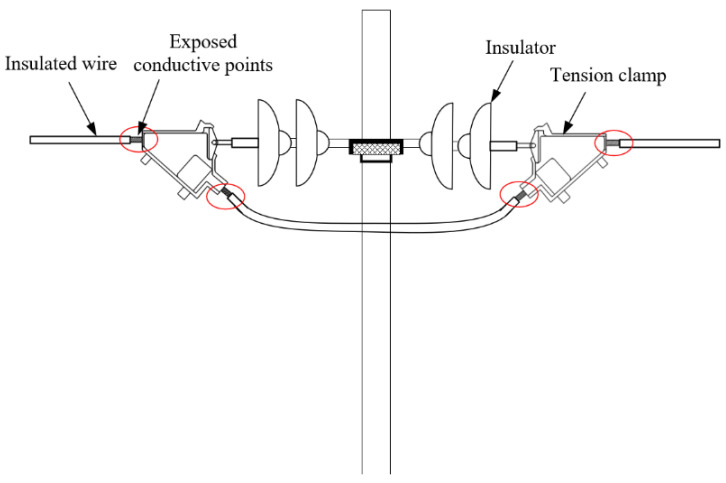
Exposed conductive points in a power grid.

With the increasing rate of bird damage accidents, bird prevention measures have been gradually developed, and can be divided into the following three kinds: (1) those relying on manual removal of bird nests and droppings: this method has a large and repetitive workload, and there is a risk of electrocution; (2) the use of bird repellent equipment to prevent and control bird damage. Commonly used repellent equipment include baffles and repellers [6]. The installation of bird repellent equipment can effectively eliminate bird damage, but birds are incredibly adaptable; (3) the use of insulated wires. Insulated wires can significantly reduce the probability of accidents caused by bird nests and droppings. However, as shown in Figure 1, near the pole/tower, due to the need for wire splicing and fixing, the wire is exposed in the tension-resistant clamp, fittings, jumpers, etc. No suitable insulation coating method has been developed for these parts.

Using epoxy resin to coat the exposed part of the insulation is an effective method to reduce outages caused by bird damage. The coating resin needs to have the characteristics of fast curing at room temperature, strong adhesion, good toughness, and strong insulation in order to meet the process requirements for insulation coating. Bisphenol A epoxy resins (DGEBA) are the most widely used matrices for engineering applications. However, as a thermosetting resin, its internal polymer chain cross-linked structure is a three-dimensional cross-linked network, resulting in a lack of toughness. Highly elastic, wear-resistant polyurethane (PU) can be used to modify epoxy resins, improving their toughness. A polythiol curing agent contains two or more sulfhydryl groups (-SH), which can rapidly cure epoxy resins at room temperature [7,8,9]. Still, an epoxy resin cured with a polythiol agent is brittle and has poor electrical properties. When epoxy resin cured at room temperature is applied to local insulation, it is influenced by the wind and the operating conditions, which places higher requirements on the mechanical and electrical properties of the resin system. A combination of polymers and fillers was reported to enhance not only the mechanical properties, thermal conductivity, and electrical properties of composites, but also their aging resistance [10,11,12,13]. Commonly used fillers include ceramic (Al_2_O_3_, BN, SiO_2_) and carbonaceous (graphene and carbon nanotubes) particles [14,15,16,17,18,19]. The toughening of room temperature curing epoxy resins with rigid particles can improve the mechanical and electrical properties of a resin system, making it more suitable for us as a coating of exposed parts of lines or parts with local insulation damage. At present, few studies have reported on the toughening of room temperature curing epoxy resin systems, and the use of micro and nanoparticles to synergistically modify resins deserves further study. In this paper, orthogonal experiments were designed for a room temperature curing system consisting of a polyurethane modified epoxy resin and a polythiol curing agent. Three particles, i.e., Al_2_O_3_, SiO_2_, and Mg(OH)_2_, were used to modify the resin matrix. The all-around performance of the prepared samples was tested to study the effect of the filler content on the performance of the epoxy resin and to provide a theoretical basis for the engineering application of room temperature curing epoxy resin.

## 2. Materials and Methods

### 2.1. Specimen Preparation

#### 2.1.1. Materials

Polyurethane-modified epoxy resin (GE-51) was provided by Chuzhou Huisheng Electronic Materials Company (Chuzhou, China). The polythiol curing agent (LN-20) was supplied by Chuzhou Huisheng Electronic Materials Company. Benzyl alcohol diluent was supplied by Guangzhou Suixin Chemical Co., Ltd. (Guangzhou, China). Nano-Al_2_O_3_ (≈20 nm) and nano-SiO_2_ (≈20 nm) were obtained from Nanjing Bokote New Materials Co. (Nanjing, China). Micron-Mg(OH)_2_ (≈10 μm) was purchased from Shandong Xinzehui New Material Technology Co. (Dongying, China).

#### 2.1.2. Specimen Preparation

**Figure 2:**
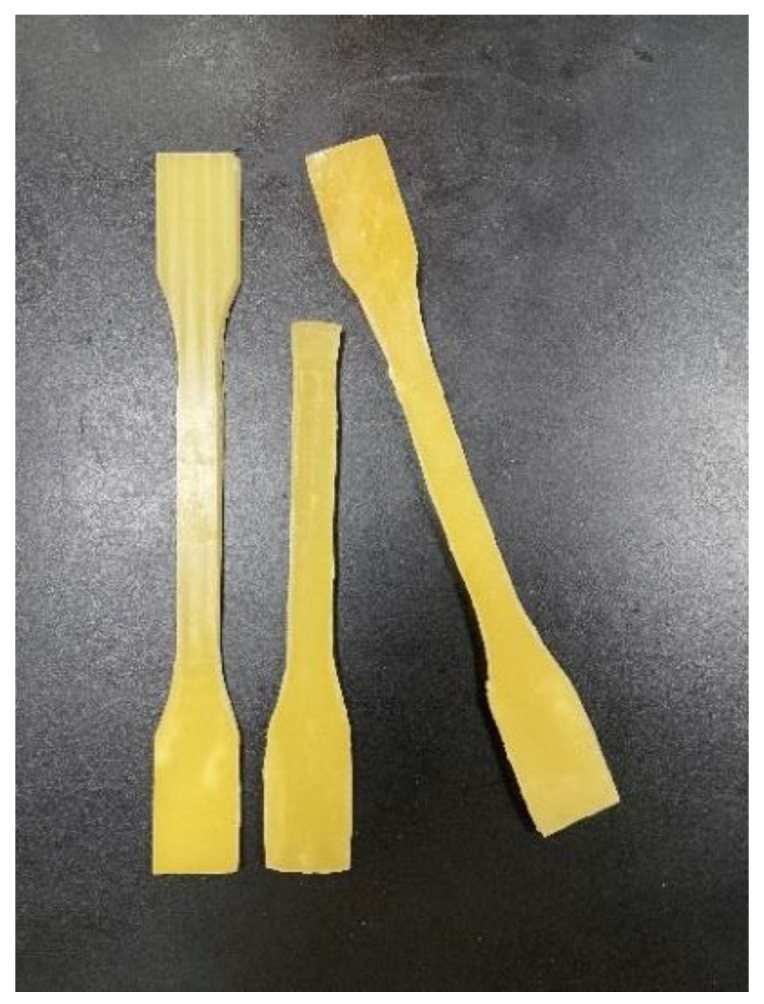
Dumbbell-shaped samples of PU-ER.

A mixture of resin, curing agent, and diluent, at a ratio of 100:70:15, was weighed. Then, three kinds of filler were weighed. The sample preparation steps were as follows: first, the resin was mixed with the diluent, and the filler and polythiol curing agent were added one after another. Then, the mixture was continuously mechanically stirred at a speed of 1000 r/min. Next, the mixed solution was vacuum defoamed at 1200 r/min and 96 kpa for 3 min using a vacuum planetary stirrer (MT-300, Mellish, Shenzhen, China). Last, the defoamed mixture was injected into a mold and cured at room temperature. The obtained samples are shown in Figure 2.

The effect of the amount of each filler in the epoxy resin system on its comprehensive performance was investigated using the orthogonal experimental method. Nano-Al_2_O_3_(A), nano-SiO_2_(B), and micron-Mg(OH)_2_(C) were used as fillers. The mass fraction of each filler relative to the PU-ER was used as the level of the factors. The resulting orthogonal factor levels are shown in Table 1, and the orthogonal experimental table is shown in Table 2:

### 2.2. Testing and Characterization

#### 2.2.1. SEM and EDS Analysis

The microscopic morphology of the specimens was observed using a scanning electron microscope (SEM: EVO MA 10/LS, Carl Zeiss Jena, Jena, Germany) with an operating voltage of 5 kV. The fracture surfaces of the tensile specimens were gold sprayed before observation. The composition of the sample elements was characterized using X-ray energy spectrometry (EDS: X-MAX, Oxford Instruments, Bognor Regis, UK) with an operating voltage of 20 kV.

#### 2.2.2. DMA Analysis

The thermomechanical properties of the thin films (35 mm × 10 mm × 1 mm) were tested using a Q800DMA (TA Instruments, New Castle, DE, USA) in single rotary arm mode and at a frequency of 10 Hz, an amplitude of 10μm, a temperature range of 50–200 °C, and a temperature rise rate of 10 °C/min.

#### 2.2.3. Thermogravimetric Analysis

Thermogravimetric analysis (TGA) is a typical means by which to analyze the thermal stability of substances. The cured polyurethane modified epoxy resin was fully ground into fine particles. Samples of 5–10 mg were tested with a thermal weight loss analyzer in a nitrogen atmosphere with a nitrogen gas flow rate of 50 mL/min and a heating rate of 10 °C/min. The test temperature range was 30–800 °C.

#### 2.2.4. Tensile Test

Using a universal testing machine to test the tensile performance of PU-ER, a tensile test following the requirements of the standard ISO527-2-2012 was undertaken. The medium width of the specimen was 10 mm, the standard distance was 50 mm, and the stretching speed was 50 mm/min.

#### 2.2.5. Water Absorption Test

The cured PU-ER samples were dried in an oven at 50 °C for 24 h and then cooled to room temperature. The sample was weighed using an analytical balance and recorded as m_1_ (accurate to 0.001 g). The sample was then completely submerged in a sufficient amount of distilled water and was weighed every 24 h; values were recorded as m_2_. The sample was dried with filter paper before weighing. The water absorption of the sample was calculated using the following formula:

(1) $$\omega =\frac{m_{2}-m_{1}}{m_{1}}$$

#### 2.2.6. Electrical Performance Test

Breakdown strength test: According to the GB/T1408.1-2016 test standard, the breakdown strength of the sample under the action of an electric field was measured at a boost rate of 1 kV/s. The measurements were taken at room temperature. The test sample had to be square in shape with a thickness of 1 mm and a width of 1000 mm. Each sample was tested ten times. The breakdown strength of different samples was analyzed using the Weibull distribution function.

Dielectric loss test: a YG9100 automatic anti-interference precision dielectric loss tester (Shanghai Yanggao Electric Co., Ltd. (Shanghai, China)) was employed to measure the dielectric loss factor. The test frequency was 50 Hz and the temperature was 25 °C. The thickness of the sample was 3 mm.

Leakage current test: According to the experimental requirements of DL/T1580-2016, the test sample was a cylinder of 60 mm in diameter and 30 mm in height. The sample was placed between two electrodes and clamped before starting the tests. The test voltage increased from 0 to 12 kV at a rate of 2 kV/s and the leakage current of the sample at 12 kV was recorded.

## 3. Formulation Optimization

### 3.1. Effect of Various Factors on the Mechanical and Physical Properties of PU-ER

**Figure 3:**
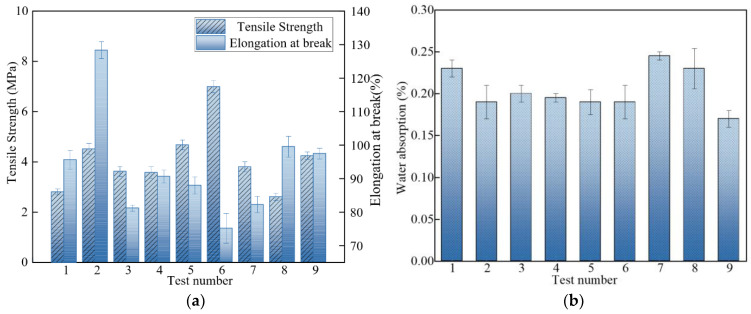
Mechanical and physical properties of different groups of PU-ER: (**a**) Tensile strength and elongation at break; (**b**) Water absorption.

The performance test results for different combinations are shown in Figure 3.

#### 3.1.1. Effect of Factors on the Tensile Strength of PU-ER

Table 3 shows the mean and extreme difference in tensile strength at different levels of each factor. From Table 3, it can be seen that the degree of influence of each factor on the tensile strength was B > A > C. The tensile strength of PU-ER increased continuously with the increasing fraction of nano-SiO_2_, from 3.40 MPa to 4.96 MPa. When the amount of SiO_2_ was 1.70%, the tensile strength was the maximum, at 4.96 MPa, i.e., 98.40% higher than that of the cured products without filler (The test results for the pure resin are in Table A1). Nano-SiO_2_ has significant surface activity and can react with the resin matrix to improve the interfacial bonds. In this way, the tensile strength of the resin matrix is significantly improved [20]. When external forces crack the system, nano-SiO_2_ plays a role in the resin matrix to hinder the further expansion of micro cracks via the silver grain mechanism.

The tensile strength of PU-ER increased and then decreased with an increasing mass fraction of nano-Al_2_O_3_. The maximum tensile strength of 5.08 MPa was obtained when the amount of alumina was 2.00%, which was 103.20% higher compared to the cured products without filler. Nano-Al_2_O_3_ also improves the tensile strength of PU-ER cured compounds due to the silver grain mechanism. In the absence of alumina nanoparticles, the resin matrix is subjected to internal and external stresses, and the silver pattern formed can further develop into destructive cracks. However, well-dispersed nanoparticles can form a “filamentary structure” with molecular chains and transform the cracks into silver patterns, which require tremendous stress to fracture the material, thus improving the tensile strength of the material. Nevertheless, when the quantity of nano-alumina particles is too large, agglomerates often form. When the volume of the agglomerates is too large and exceeds the void inside the crack, the nano-alumina particles cannot enter to cause the crack transform into a silver pattern, resulting in reduced tensile strength.

It can be seen from Table 3 that the tensile strength of PU-ER gradually decreased from 4.14 MPa to 4.04 MPa as the mass fraction of Mg(OH)_2_ increased. With the addition of Mg(OH)_2_, the larger Mg(OH)_2_ particles introduced more and more weak interfaces, and the number of two-phase defects kept increasing, leading to a decrease in tensile strength [21]. However, with an increasing number of Mg(OH)_2_ particles, the smaller Al_2_O_3_, and SiO_2_ particles could eliminate the defects formed by Mg(OH)_2_ particles to some extent and form a tight structure with the resin matrix, which was the reason for the smaller decrease in tensile strength.

#### 3.1.2. Effect of Various Factors on Elongation at Break of PU-ER

Elongation at break is a measure of the toughness. Table 4 shows the mean and extreme difference of elongation at break for different levels of each factor. It can be seen that the degree of influence of each factor on the water absorption is C > B > A. As the mass fraction of nano-Al_2_O_3_ increased, the elongation at break of PU-ER first decreased and then increased. The maximum value was 101.76% when the mass fraction of nano-Al_2_O_3_ was 1.5%. The amount of SiO_2_ and Mg(OH)_2_ had the same effect on this parameter. The elongation at the break of PU-ER increased first and then decreased with an increasing amount of SiO_2_ and Mg(OH)_2_. The maximum values for PU-ER were 105.33% and 105.86% when the fractions of SiO_2_ and Mg(OH)_2_ were 1% and 7%, respectively.

#### 3.1.3. Effect of Various Factors on the Water Absorption of PU-ER

In order to effectively enhance the local insulation at wire clips, fixtures, and jumpers in a distribution network, there are requirements regarding the water absorption rate of the insulation material. The water absorption of composite materials is related to many factors such as pores and impurities at the interface of resin and filler [22,23]. Table 5 shows the mean and extreme difference in water absorption rates for each factor. It can be seen that the degree of influence of each factor on water absorption was B > A > C, from large to small. The water absorption of PU-ER gradually decreased as the mass fraction of SiO_2_ and Mg(OH)_2_ increased. The addition of particles eliminated the defects in the resin network, while the close bonding of particles with the resin matrix eliminated some of the voids. With an increase of Al_2_O_3_, the water absorption of the cured material first increased and then decreased. When the mass fraction of Al_2_O_3_ was 2%, the lowest water absorption of the composite was 0.19%. This was because the filler distribution was more uniform when the mass fraction of Al_2_O_3_ was 2%, whereby it could minimize minor defects in the cross-linked network of resin. When the mass fraction of Al_2_O_3_ was 2.5%, the negative effect of filler particle agglomeration exceeded the positive effect of particle filling defects, and the water absorption increased [24].

### 3.2. Effect of Various Factors on the Electrical Performance of PU-ER

**Figure 4:**
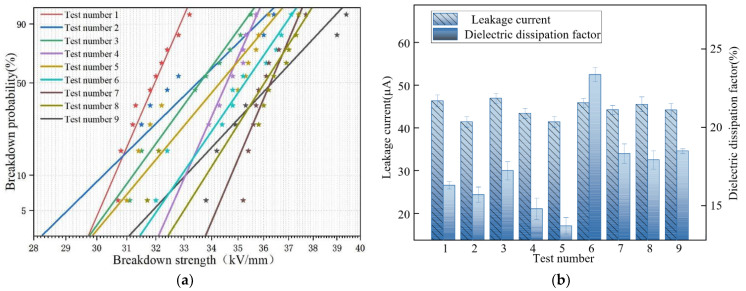
Electrical properties of different groups of PU-ER: (**a**) Breakdown strength; (**b**) Leakage current and dielectric loss factor.

The results of electrical performance tests for different combinations are shown in Figure 4:

#### 3.2.1. Effect of Various Factors on the Industrial Frequency Breakdown Strength of PU-ER

Breakdown strength is one of the significant electrical property indexes of insulating materials, reflecting the material’s electrical resistance. Figure 4a shows the Weibull probability distribution of breakdown strength of different combinations of PU-ER. It can be seen that the breakdown strength of the cured material was more scattered with different doping amounts. Table 6 shows the mean and extreme deviation of the breakdown strength at different levels of working frequency for each factor. As shown, the degree of influence of each factor on the breakdown strength was A > B > C. The breakdown strength of PU-ER increased with an increase of nano-Al_2_O_3_ and nano-SiO_2_. When nanoparticles were added to the resin, the resin displayed a higher breakdown voltage because its enormous specific surface reduced the inherent faults in the resin. In contrast, the nanoparticles themselves did not introduce defects. When the amount of nano-Al_2_O_3_ was low, some of the nanoparticles were able to remove the flaws in the polymer. However, the nanoparticles connected the surrounding polymer molecules very tightly. They broke the connections between the polymer molecules around the nanoparticles and the distant polymer molecules, which resulted in numerous new flaws, occurring like isolated “islands” [25]. When high voltage was applied to the epoxy composite, electrons were more inclined to break through from around the defects. When the nanoparticle content was high, the original “islands” could be linked by the nano-alumina particles to form a tight structure, and the defects were largely eliminated, improving the breakdown strength of the composite. Due to the large particle size and small specific surface area of micron particles, doping in a resin does not result in a tight structure with the base material. However, it will introduce more defects, which might decrease the breakdown strength [26,27]. However, we found that nanoparticles could eliminate some defects when the micron particle content was negligible. Therefore, as the micron-Mg(OH)_2_ content increased, the breakdown strength first increased and then decreased.

#### 3.2.2. Effect of Various Factors on PU-ER Leakage Current 

Measuring the leakage current is an effective method of evaluating the interfacial properties of a resin. Table 7 shows each the mean and extreme difference regarding leakage current at different levels for each factor. The degree of influence of each factor on the leakage current was shown to be B > C > A. The leakage current of the cured material decreased and then increased with an increase of nanoparticle content. Nanoparticles were able to form a tight connection between macromolecules while eliminating resin defects, thereby hindering the movement of electrons. This was the reason why the leakage current decreased initially. However, as the nanoparticle content increased, the particle agglomeration phenomenon led to another increase in the leakage current. For micron Mg(OH)_2_, which had a smaller specific surface area than nanoparticles, the particles could not eliminate defects in the resin as the nanoparticles did, and defects were introduced by the micron particles themselves, which led to more significant leakage currents [28]. Therefore, an increase of micron-Mg(OH)_2_ content caused the leakage current to increase. However, as mentioned earlier, when the micron-Mg(OH)_2_ content was low, the nanoparticles could remove some of the defects introduced by the defective micron-Mg(OH)_2_, resulting in a slight decrease in the leakage current.

#### 3.2.3. Effect of Various Factors on Dielectric Loss of PU-ER

Dielectric loss not only consumes electrical energy but also makes the components heat up and affects their performance. Table 8 shows the mean and extreme difference of the dielectric loss factor for each factor at different levels. As shown, the degree of influence of each factor was B > C > A. With an increase of the mass fraction of nano-SiO_2_, the tanδ first decreased and then increases. The dispersion in the resin was uniform when the percentage of SiO_2_ was lower. Large interfacial layers were created by the high specific surface area, which strengthened the bond between the nanoparticles and resin matrix. The binding of carriers inhibited the material polarization, so the tanδ decreased. With an increase of SiO_2_, the dispersion of the particles decreased, encouraging the overlapping of the interfacial layers. Weaker binding of carriers by the resin in the overlapping region weakened the inhibition of the polarization process and caused tanδ to increase [29]. In contrast, due to their relatively greater nano-Al_2_O_3_ content, nanoparticles underwent agglomeration at a content of 1.5%, leading to interfacial layer overlap and a continuous decrease in tanδ [30,31,32]. Compared to nanoparticles, micron particles have smaller specific surface areas, and the interfacial effect that hindered the medium polarization was not apparent after micron particle doping. An increase of Mg(OH)_2_ enhanced the polarization behavior of the composites to some extent. Nevertheless, the larger molecular size of micron particles made the polymer matrix more viscous, which could inhibit the polarization of charged particles and reduce the dielectric loss of such a resin system [33].

### 3.3. Determination of the Optimal Formulation Combination

The results of orthogonal experiments showed that the amount of nano-SiO_2_ was the main factor affecting the tensile strength, water absorption, leakage current, and dielectric loss factor. The improvement of mechanical properties and water absorption was the primary objective; leakage current and dielectric loss were secondary considerations, and as such, the optimal amount of nano-SiO_2_ was determined to be 1.7%.

The amount of nano-Al_2_O_3_ was the first factor affecting the breakdown strength and the second one affecting tensile strength. The breakdown strength of PU-ER showed that the resin matrix could effectively enhance the local insulation for a long time. As such, the optimal amount of nano-Al_2_O_3_ was determined to be 2.5%.

Micron-Mg(OH)_2_ is the first factor affecting the elongation at break and the second-one affecting dielectric loss and leakage current. The optimal amount of micron-Mg(OH)_2_ was found to be 7%. In summary, the optimal formulation of the room temperature curing epoxy resin was determined to be A_3_B_3_C_2_. The tensile strength of PU-ER was 4.25 MPa, the elongation at break was 97.5%, and the breakdown strength was 36.9 kV/mm.

## 4. Discussion and Analysis of Results

After studying the effects of different combinations on the mechanical and electrical properties of PU-ER via orthogonal tests, the optimal formulation of the resin system (A_3_B_3_C_2_) was determined. The effects of fillers on the microscopic morphology and thermal properties of the resin system were also investigated by SEM, EDS, and DMA. Finally, TGA tests were carried out when the formulation was A_3_B_3_C_2_.

### 4.1. Micromorphological Analysis

**Figure 5:**
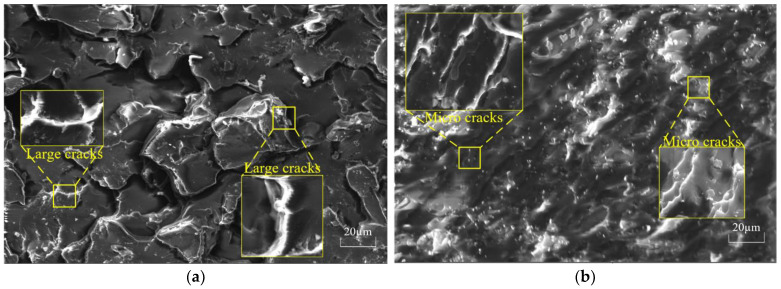
SEM diagram of tensile section of resin before and after adding filler: (**a**) Pure resin; (**b**) Resin after adding filler.

SEM can be used to study the surface morphologies of resin matrices. Figure 4 shows the tensile section of the resin matrix before and after doping with filler under SEM. From Figure 5a, it can be seen that the resin matrix without filler was more continuous and locally smoother with fewer cracks but with brittle fractures. In comparison, more cracks appeared on the resin matrix shown in Figure 5b because the addition of filler particles caused the resin matrix and the filler particles to combine more effectively. When the resin matrix was stressed, the structure formed by the particles and the resin matrix made the resin fracture only after a lot of deformation, i.e., a ductile fracture [22,34].

**Figure 6:**
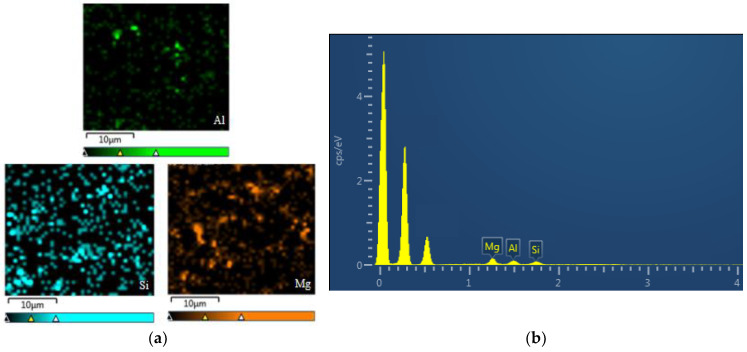
Tensile cross-sectional EDS diagram after doping with filler: (**a**) Distribution diagram of different elements; (**b**) EDS spectra.

The wavelength and intensity of the characteristic X-rays produced by the interaction of electrons with the specimen are measured by X-ray energy spectrometry (EDS), enabling researchers to carry out qualitative or quantitative analyses of the elements contained in minute regions. Figure 6 shows the EDS spectrum of the resin matrix after doping with filler. From Figure 5, it can be seen that the Al, Si, and Mg were more uniformly dispersed in the resin matrix.

### 4.2. Thermomechanical Property Analysis

**Figure 7:**
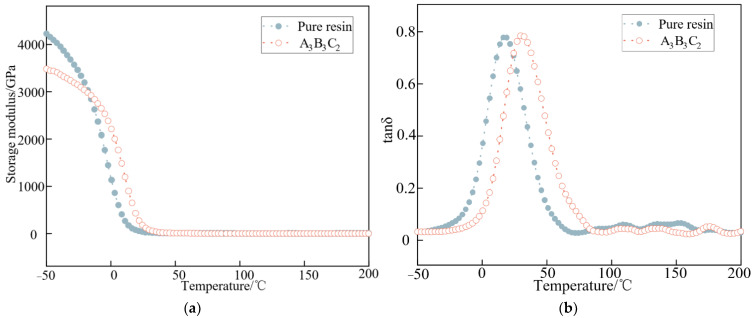
Storage modulus-temperature spectrum and loss factor−temperature spectrum of different groups of PU-ER: (**a**) Storage modulus-temperature spectrum; (**b**) Loss factor−temperature spectrum.

The dynamic mechanical properties of composites are critical performance indicators which may be used to characterize the viscoelasticity of resin matrix composites and the interaction between the nano-reinforcements and the resin matrix. The curves of the energy storage modulus (E’) and loss angle tangent (tanδ) versus temperature for different combinations are shown in Figure 7. The dynamic energy storage modulus of PU-ER decreased with an increase of temperature. Before the glass transition temperature was reached, the cured material was in a glassy state. At the same time, the movement of molecular chain segments was restricted, and only the stretching vibration of structural groups existed. The cured material exhibited rigid properties, and the energy storage modulus was high. As the temperature continued to increase, the cured material entered a rubbery state, in which the movement of molecular chain segments was enhanced, and the energy storage modulus decreased [35]. From Figure 7a, it can be seen that the energy storage modulus of PU-ER decreased due to the addition of filler. The filler was involved in the cross-linking reaction of the resin, and the reduction of the cross-linking density of the system enhanced the molecular chain segment movement, resulting in a reduction of the energy storage modulus of the cured material. All systems showed a peak in the loss factor tanδ and only one T_g_. From Figure 7b, it can be seen that the addition of filler caused a right shift in the tanδ curve. The glass transition temperature of the resin before and after the addition of filler is shown in Table 9. As shown, the T_g_ of the resin increased from 17.06 °C to 30.54 °C after the addition of filler. This was because the addition of particles reduced the free space between the macromolecules, which increased the stiffness of the composite [36].

### 4.3. Thermal Stability Analysis

**Figure 8:**
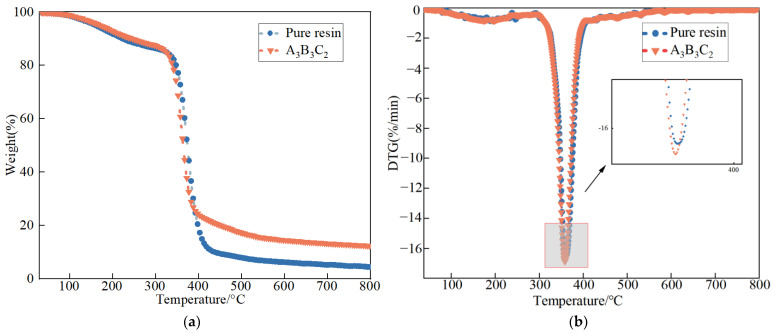
TG and DTG curves of different groups of PU-ER: (**a**) TG; (**b**) DTG.

The thermal weight loss curves of the epoxy resin and the composite could be used to study the thermal stability changes of the materials. Figure 8 shows the TG and DTG curves of PU-ER before and after the addition of the filler. The initial degradation temperature (T_5%_), halfway degradation temperature (T_50%_), and temperature at maximum weight loss rate (T_max_) of PU-ER are listed in Table 10. It can be seen that the T_5%_ of PU-ER before the addition of filler was 156.40 °C; the addition of filler resulted in an increase of T_5%_ to 169.88 °C. As mentioned, the filler was able to react with the resin matrix, resulting in a more compact cross-linked structure of the composite, which led to an increase in T5_%_. In addition, the temperature (T_max_) corresponding to the maximum rate of weight loss before and after filler addition was 359.41 °C and 358.20 °C, respectively. This indicates that the filler has little effect on the overall thermal stability of the resin system [37,38]. The degradation behavior exhibited by the composites during thermal decomposition was similar to that of the pure resin, indicating that the introduction of the filler did not significantly change the degradation mechanism of the resin itself.

## 5. Conclusions

In this paper, the physicochemical and electrical properties of PU-ER modified by different fillers were investigated. The effects of different particles on the comprehensive properties of PU-ER and the synergistic effects of three particles were analyzed by designing orthogonal experiments. The amount of nano-Al_2_O_3_ was shown to be the main factor affecting the breakdown strength and the second factor affecting the tensile strength. The amount of nano-SiO_2_ was the main factor affecting tensile strength, water absorption, leakage current, and dielectric loss factor, while micron Mg(OH)_2_ was the main factor affecting elongation at break. When multiple fillers were compounded to modify the resin, the effect of each particle-modified resin was still reflected, while a synergistic effect of micro and nanoparticle modification emerged. The smaller particle size of nano-Al_2_O_3_ and nano-SiO_2_ could fill the voids formed between Mg(OH)_2_, forming a relatively tight structure and increasing the tensile strength. The optimal formulation of the room temperature curing epoxy resin is A_3_B_3_C_2_. The tensile strength of A_3_B_3_C_2_ is 4.25 MPa, the elongation at break is 97.5%, and the breakdown strength is 36.9 kV/mm. In addition, the microscopic morphology and thermal properties of PU-ER were investigated when the filler formulation was A_3_B_3_C_2_. SEM and EDS tests showed that the filler could bind to the resin matrix and generate more cracks to absorb energy when subjected to stress. DMA tests showed that adding the filler reduced the free space between the macromolecules, leading to an increase in the stiffness of the composite and in the glass transition temperature of the cured material. TGA tests showed that the introduction of fillers did not significantly change the degradation mechanism of the resin and increased its T5%. In conclusion, the composite had the best overall performance when the amounts of SiO_2_, Al_2_O_3_, and Mg(OH)_2_ were 1.7%, 2.5%, and 7%, respectively.

## Figures and Tables

**Table 1:** Orthogonal experimental factor levels.

Level	Factor		
	Al_2_O_3_/%	SiO_2_/%	Mg(OH)_2_/%
1	1.5	0.3	3
2	2	1	7
3	2.5	1.7	11

**Table 2:** Specific experimental design schemes in the orthogonal experiment.

Test Number	Project	Level		
		Al_2_O_3_/%	SiO_2_/%	Mg(OH)_2_/%
1	A_1_B_1_C_1_	1.5	0.3	3
2	A_1_B_2_C_2_	1.5	1	7
3	A_1_B_3_C_3_	1.5	1.7	11
4	A_2_B_1_C_2_	2	0.3	7
5	A_2_B_2_C_3_	2	1	11
6	A_2_B_3_C_1_	2	1.7	3
7	A_3_B_1_C_3_	2.5	0.3	11
8	A_3_B_2_C_1_	2.5	1	3
9	A_3_B_3_C_2_	2.5	1.7	7

**Table 3:** Mean and extreme difference analysis of tensile strength for each factor (MPa).

Project	Factor		
	Al_2_O_3_	SiO_2_	Mg(OH)_2_
Mean of level 1	3.65	3.40	4.14
Mean of level 2	5.08	3.94	4.11
Mean of level 3	3.56	4.96	4.04
Extreme difference	1.52	1.56	0.10

**Table 4:** Mean and extreme difference analysis of elongation at break for each factor (%).

Project	Factor		
	Al_2_O_3_	SiO_2_	Mg(OH)_2_
Mean of level 1	101.76	89.57	90.17
Mean of level 2	84.66	105.33	105.86
Mean of level 3	93.14	84.66	83.87
Extreme difference	17.10	20.67	21.99

**Table 5:** Mean and extreme difference analysis of water absorption for each factor (%).

Project	Factor		
	Al_2_O_3_	SiO_2_	Mg(OH)_2_
Mean of level 1	0.21	0.22	0.22
Mean of level 2	0.19	0.20	0.21
Mean of level 3	0.22	0.18	0.21
Extreme difference	0.03	0.04	0.01

**Table 6:** Mean and extreme difference analysis of the breakdown voltage of each factor (kV/mm).

Project	Factor		
	Al_2_O_3_	SiO_2_	Mg(OH)_2_
Mean of level 1	33.5	34.47	34.67
Mean of level 2	35.13	35.17	35.4
Mean of level 3	36.6	35.6	35.17
Extreme difference	3.1	1.13	0.73

**Table 7:** Mean and extreme difference analysis of leakage current of each factor (μA).

Project	Factor		
	Al_2_O_3_	SiO_2_	Mg(OH)_2_
Mean of level 1	44.913	44.675	44
Mean of level 2	44.257	43.14	44.531
Mean of level 3	44.995	46.35	45.63
Extreme difference	9.783	3.21	1.63

**Table 8:** Mean and extreme difference analysis of dielectric loss of each factor (%).

Project	Factor		
	Al_2_O_3_	SiO_2_	Mg(OH)_2_
Mean of level 1	16.42	16.495	16.177
Mean of level 2	17.3	15.79	19.14
Mean of level 3	18.26	19.705	16.66
Extreme difference	1.84	3.915	2.963

**Table 9:** Glass transition temperature of resin before and after adding filler.

Project	T_g_ (°C)
Pure resin	17.06
A_3_B_3_C_2_	30.54

**Table 10:** TGA parameters before and after adding filler/ °C.

Project	T_5%_ (°C)	T_50%_ (°C)	T_max_ (°C)
Pure resin	156.40	373.04	359.41
A_3_B_3_C_2_	170.44	363.38	358.20

## Data Availability

Not applicable.

## Appendix A

**Table A1:** Performance test results of PU-ER without filler.

PU-ER without Filler	
Tensile strength (MPa)	2.50
Elongation at break (%)	58.20
Water absorption rate(%)	0.17
Breakdown strength (kV/mm)	30.67
Leakage current (µA)	50.12
Dielectric loss (%)	25.44
